# Elevated cerebral spinal fluid biomarkers in children with mucopolysaccharidosis I-H

**DOI:** 10.1038/srep38305

**Published:** 2016-12-02

**Authors:** Gerald V. Raymond, Marzia Pasquali, Lynda E. Polgreen, Patricia I. Dickson, Weston P. Miller, Paul J. Orchard, Troy C. Lund

**Affiliations:** 1 Division of Pediatric Neurology, University of Minnesota, Minneapolis, MN, USA; 2 University of Utah, School of Medicine, Department of Pathology, Salt Lake City, UT, USA; 3 Los Angeles Biomedical Research Institute at Harbor-UCLA Medical Center, Los Angeles, CA, USA; 4 Division of Pediatric Blood and Marrow Transplant, University of Minnesota, Minneapolis, MN, USA.

## Abstract

Mucopolysaccharidosis (MPS) type-IH is a lysosomal storage disease that results from mutations in the *IDUA* gene causing the accumulation of glycosaminoglycans (GAGs). Historically, children with the severe phenotype, MPS-IH (Hurler syndrome) develop progressive neurodegeneration with death in the first decade due to cardio-pulmonary complications. New data suggest that inflammation may play a role in MPS pathophysiology. To date there is almost no information on the pathophysiologic changes within the cerebral spinal fluid (CSF) of these patients. We evaluated the CSF of 25 consecutive patients with MPS-IH. While CSF glucose and total protein were within the normal range, we found a significantly mean elevated CSF opening pressure at 24 cm H_2_O (range 14–37 cm H_2_O). We observed a 3-fold elevation in CSF heparan sulfate and a 3–8 fold increase in MPS-IH specific non-reducing ends, I0S0 and I0S6. Cytokine analyses in CSF of children with MPS-IH showed significantly elevated inflammatory markers including: MCP-1 SDF-1a, IL-Ra, MIP-1b, IL-8, and VEGF in comparison to unaffected children. This is the largest report of CSF characteristics in children with MPS-IH. Identification of key biomarkers may provide further insight into the inflammatory-mediated mechanisms related to MPS diseases and perhaps lead to improved targeted therapies.

Severe mucopolysaccharidosis type I, Hurler’s syndrome (MPS-IH), is a lysosomal storage disease due to mutations in the *IDUA* gene resulting in decreased/absent alpha-L-iduronidase activity. The consequent accumulation of the glycosaminoglycans (GAGs), heparan sulfate (HS) and dermatan sulfate (DS), in tissues results in a number of clinical features including hepatosplenomegaly, progressive cognitive impairment, cardiovascular complications, and joint and bone abnormalities (dysostosis multiplex)^1^. Currently, exogenous enzyme replacement using recombinant alpha-L-iduronidase is available to patients with MPS-I, although it does not cross the blood brain barrier in significant amounts^2^.

To achieve continuous enzyme delivery as well as provide a cerebral source of cells expressing alpha-L-iduronidase (presumed to be microglia), hematopoietic cell transplant (HCT) is used as standard of care for patients with Hurler syndrome^3^,^4^. HCT leads to an increase in IDUA enzyme activity and concomitant reductions in substrate levels as well as stabilization of neurodegeneration^5^,^6^,^7^. HCT does not arrest the progression of joint and bone disease^8^,^9^,^10^,^11^, nor does it reverse the characteristic changes in the heart valves^12^,^13^. These observations suggest that GAG accumulation is not the sole mediator of disease-related complications in MPS-IH^14^. Recent work in rodent models supports that co-existent immune and microglial inflammatory processes contribute to the pathology of several MPS diseases with demonstrating several key inflammatory cytokines including IL-6, IL-8, MIP1-beta, MIP1-alpha, and MCP-1^15^,^16^,^17^.

As mentioned, prior to the development of HCT, children with MPS-IH were observed to develop progressive, debilitating developmental and cognitive deterioration^7^. While there have been several descriptions of various plasma biomarkers for MPS-IH^5^,^18^,^19^,^20^,^21^, no study has systematically evaluated the cerebrospinal fluid (CSF). Here, we document for the first time, the characteristics of MPS-IH CSF with a focus on inflammatory cytokines.

## Results

**Figure 1:**
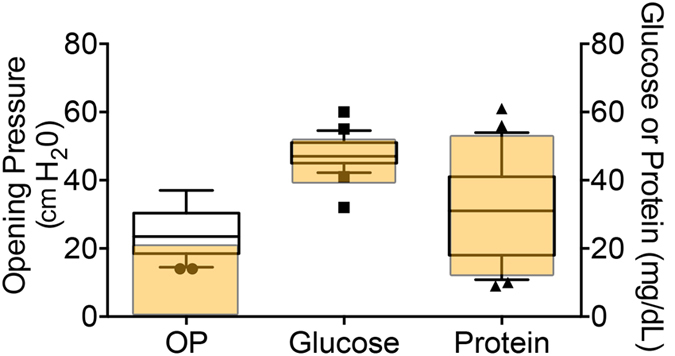
CSF opening pressure (OP), glucose, and total protein in MPS-IH patients. The boxes represent to 25^th^ to 75th percentiles with a line at the mean. Whiskers show the 10^th^ and 90^th^ percentiles. Symbols represent value outside the 10 – 90^th^ percentile. The yellow area indicates the normal range for age at our institution.

We performed a lumbar puncture and CSF analysis on 25 consecutive patients with MPS-IH with a median age of 11.2 months. Nearly all CSF was free from erythrocytes or white blood cells. As shown in Fig. 1, the mean CSF glucose concentration was 47.6 (range 32.2–60.1 mg/dL), with the normal range for age being 40–70 mg/dL. The mean CSF total protein was 30.8 (range 9.3–61.6 mg/dL), with a normal range for age of 15–60 mg/dL. Strikingly, we found a significant elevation in opening pressure (OP) in children with MPS-IH, with a mean of 24.6 cm H_2_O (range 14–37 cm H_2_O). This is higher than what is considered a normal OP in children of this age, which is <20 cm H_2_O^22^.

**Figure 2:**
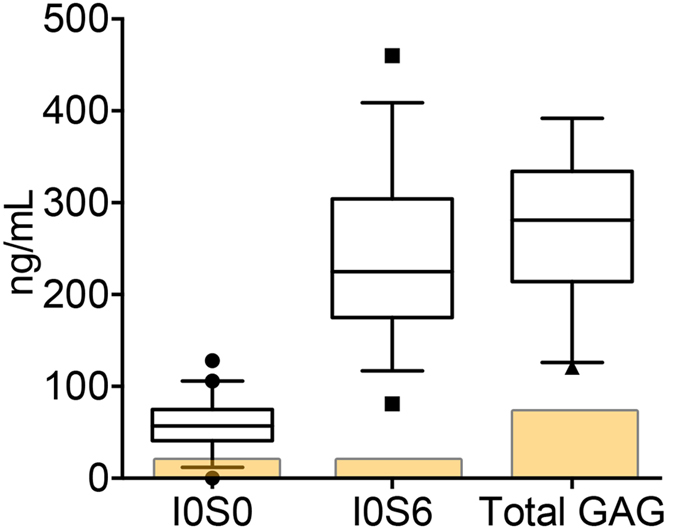
Non-reducing ends (NRE)s, I0S0 and I0S6, and total HS content in MPS-IH CSF. The boxes represent to 25^th^ to 75th percentiles with a line at the mean. Whiskers show the 10^th^ and 90^th^ percentiles. Symbols represent value outside the 10 – 90^th^ percentile. The yellow area indicates the normal range in the general population.

Using the Sensi-Pro ^®^ assay, we measured NREs characteristic for MPS-IH, I0S0 and I0S6, and also determined total HS concentration^19^. We found a significant elevation in I0S0 and I0S6 with an average of 56.3 and 249.1 ng/mL respectively (with normal values of <15 and <30 ng/mL, respectively^19^) as shown in Fig. 2. Total HS was also significantly elevated with an average of 278.1 ± 108.2 ng/mL (normal <120 ng/mL) (Fig. 2).

**Figure 3:**
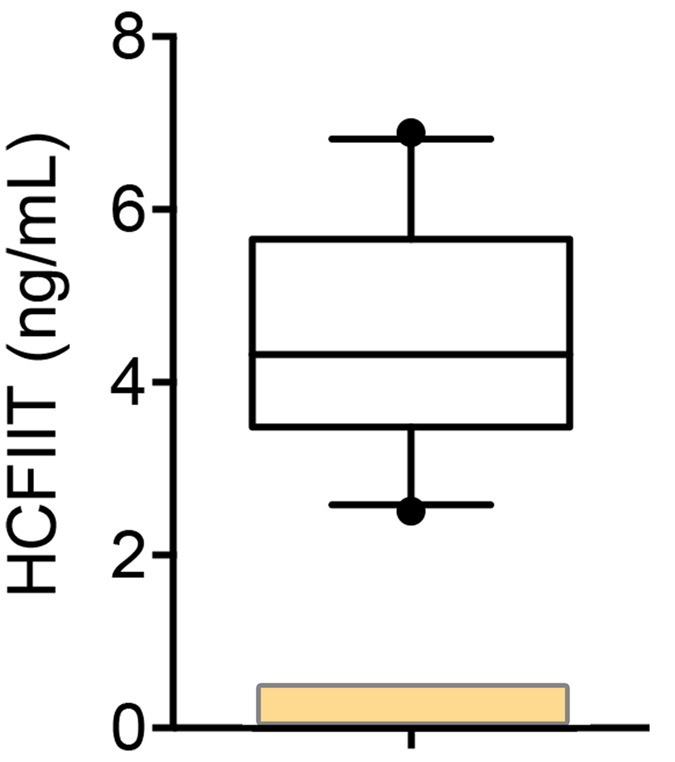
Levels of HCII-T in MPS-IH CSF. The boxes represent to 25^th^ to 75th percentiles with a line at the mean. Whiskers show the 10^th^ and 90^th^ percentiles. Symbols represent value outside the 10 – 90^th^ percentile. The yellow area indicates the non-MPS reference. N = 10 MPS-IH patients. The reference value was determined from the average of four non-MPS samples.

We also evaluated HCII-T (heparin cofactor II-thrombin) complex, a previously described biomarker of lysosomal storage diseases including MPS-IH^18^. We found a significant elevation with a mean level of 4.5 ± 1.4 ng/mL of HCII-T complex (reference range: <0.25 ng/mL) (Fig. 3).

**Figure 4:**
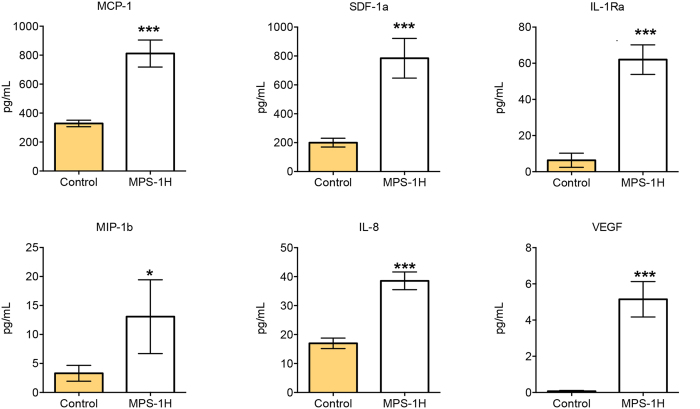
CSF Inflammatory cytokines in MPS-IH patients. Shown are the factors that demonstrated significant elevation in the MPS-IH group. Error bars represent standard error of the mean and p-values were generated from a Student’s t-test. *p < 0.05, ***p < 0.001.

We found six inflammatory markers to be significantly elevated in children with MPS-IH when compared to controls: monocyte chemoattractant protein-1 (MCP-1) (mean 811 vs 328 pg/mL, p < 0.001), stromal cell-derived factor-1a (SDF-1a) (784 vs 200 pg/mL, p < 0.0001), interleukin-1 receptor antagonist (IL-Ra) (62 vs 6 pg/mL, p < 0.0001), macrophage inflammatory protein 1-beta (MIP-1b) (13.1 vs 3.3 pg/mL, p = 0.04), interleukin-8 (IL-8) (39 vs 17 pg/mL, p < 0.0001), and vascular endothelial growth factor (VEGF) (5.1 vs 0.1 pg/mL, p < 0.0001) as shown Fig. 4.

## Discussion

We report potential CSF biomarkers for patients with MPS-IH. These may be important to consider as further therapies are being developed either through immunomodulation, hematopoietic stem cell transplant, new forms of enzyme therapy and other interventions. These biomarkers may serve as indicators to which we can compare the effectiveness of new interventions. In addition, they may prove useful as a means of identifying future phenotypes in children diagnosed through newborn screening that display novel or poorly characterized genotypes.

We found the mean CSF OP was higher in children with MPS-IH than what is considered “typical” for healthy children, which is <20 cm H_2_0^22^. Recently, Avery *et al*. analyzed the OP of 197 children and found a mean OP of 19.6 cm H_2_0^23^. Furthermore, given the 10^th^/90^th^ percentage of Avery’s data was 11.5 and 28 cm H_2_O, it has been suggested that an abnormal OP should be consider that of >28 cm H_2_O^23^,^24^. Six of 25 MPS-IH patients had OP greater than 30 cm H_2_O. While sedation and changes in ventilation can both modulate the OP^24^, our patients all had strict end-tidal CO_2_ monitored and maintained from 25–40 mm Hg. Classically, elevated OP is associated with intracranial processes such as infection (bacterial, viral, or fungal meningitis), subarachnoid hemorrhage, pseudotumor cerebri, or any communicating hydrocephalus. Our data suggest that GAG accumulation and perhaps subacute neuroinflammation may contribute to an increase in OP. We should note that none of our patients has evidence of papilledema suggesting that their increased OP was not severe enough to affect the optic nerve head.

An inflammatory process has been implicated as a pathological contributor to MPS disease^21^, with specific contributions to skeletal manifestations. Simonaro *et al*. previously found TNF-alpha to be elevated in MPS VII mice and treatment of MPS VI affected rats with Infliximab, an antibody targeted to TNF-alpha, significantly reduced joint disease^15^. Additionally, the anti-inflammatory compound, pentosan polysulfate, has been shown to reduce inflammation associated bone pathology in a rat model of MPS VII and is now entering clinical trials in MPS patients^25^.

In this study, we demonstrate for the first time that markers of inflammation are manifest in the CSF of MPS patients. Our data is consistent with what others have shown in the brain of MPS animal models. For example, Wilkinson *et al*. showed significantly high levels of MCP-1 in the brain of MPSI, MPSIIIA, and MPSIIIB mice^17^. The links between the immune system of MPS pathology as it relates to the neurological and skeletal system is becoming more appreciated^26^. It is doubtful that a single cytokine or inflammatory factor is responsible for MPS pathology, as many of the inflammatory proteins exist in a “cascade” of factors where initiation of inflammation is followed by waves of chemokine secretion and recruitment of immune cells. Several of the elevated factors we show here are also associated with other neuroinflammatory conditions, including MCP-1 and MIP-1b which are elevated in patients with multiple sclerosis, while MCP-1, MIP-1b, IL-8, and SDF-1a are elevated in stroke victims^27^.

Whether the CSF inflammatory mediators are due to GAG accumulation or another process is not known. There are very few reports of CSF GAG evaluation in patients afflicted with a mucopolysaccharidosis diagnosis^28^,^29^,^30^, and we believe this is the first study to evaluate non-reduced ends (NREs) and HS levels in the CSF of MPS-IH patients. Clinical trials investigating the use of anti-inflammatory agents are being developed in MPS-I and other MPS subtypes for the purpose of ameliorating joint and bone disease^25^. Novel attempts at targeting the CSF with recombinant viral vectors delivering the missing enzyme are being developed in several MPS diseases as well^31^,^32^,^33^,^34^,^35^. Commonly, glycan-based markers are used to show efficacy for these various strategies^36^. Based on our CSF findings, it may also be important to collect and assess both CSF GAG and inflammatory markers as new clinical trials evolve, since reducing inflammation will likely coincide with an impact on neurological processes and perhaps skeletal disease as noted above.

## Methods

### Participants

Patients with MPS-IH (n = 25, median age of 11.2 months, range 6–30 months) had CSF sampling performed 8 weeks prior to hematopoietic stem cell transplant at the University of Minnesota. During the initial evaluation including a sedated MRI, a lateral decubitus lumbar puncture is routinely performed with an opening pressure (OP) measurement and CSF was obtained and analyzed for cell count, protein concentration, glucose concentration, GAG concentration and cytokine analysis. End-tidal CO_2_ monitored and maintained from 25–40 mm Hg to ensure opening pressure accuracy.

Control patients for biomarker analyses (n = 25, median age 6.8 years, range 4–17 years) were those undergoing intrathecal chemotherapy for a prior diagnosis of acute lymphoblastic leukemia, at least 3 months into maintenance therapy, and without a CSF leukemia diagnosis. In controls, CSF was withdrawn just prior to administration of the intrathecal chemotherapy and cytokine concentrations determined by ELISA. Unavailability of “healthy” controls due to the risks inherent to attaining CSF from “healthy” children established these patients as the most appropriate control group available and has been previously published by our group and others^37^,^38^,^39^. This study was approved by the Committee on the Use of Human Subjects in Research at the University of Minnesota, and all experiments were performed in accordance with relevant guidelines and regulations by the Committees on the Use of Human Subjects in Research at the University of Minnesota. Informed written consent was obtained for all patient samples from the parents or guardians on behalf of the child participants.

### Cytokines

CSF samples were evaluated using the 22-plex, human panel A, (R&D Systems, Minneapolis, MN) measured with the Luminex system (Luminex, Austin, TX) and analyzed by Bioplex software (BioRad, Hercules, CA). This panel includes ENA-78, bFGF, G-CSF, GM-CSF, IFN-gamma, IL-1alpha, IL-1beta, IL-1ra, IL-2, IL-4, IL-5, IL-6, IL-8, IL-10, IL-17, MCP-1, MIP-1alpha, MIP-1beta, RANTES, TNF-alpha, TPO, and VEGF as shown in Table 1. SDF-1alpha was measured by sandwich ELISA (R&D Systems, Minneapolis, MN).

### Heparin cofactor II-thrombin (HCII-T)

HCII-T complex was determined by ELISA, following the manufacturer’s instruction (#MBS904277, Mybiosource, San Diego, CA).

### Non-Reducing Ends (NREs) and total HS

The CSF NREs (I0S0 and I0S6) and total HS (calculated from the addition of the internal disaccharides, D0A0 + D0S0) were determined using the Sensi-Pro ^®^ assay as previously described^19^.

### Statistical methods

Cytokine measurements were made in duplicate and the average of the two values was used to determine concentration using standard curves generated with the relevant recombinant human proteins provided with the commercial kits. Means for the MPS-IH and control groups were calculated and subjected to a two-tailed Student’s t-test to compute a p-value.

## Additional Information

**How to cite this article**: Raymond, G. V. *et al*. Elevated cerebral spinal fluid biomarkers in children with mucopolysaccharidosis I-H. *Sci. Rep.* **6**, 38305; doi: 10.1038/srep38305 (2016).

**Publisher’s note:** Springer Nature remains neutral with regard to jurisdictional claims in published maps and institutional affiliations.

## Figures and Tables

**Table 1:** List of inflammatory factors evaluated in CSF samples.

Cytokine Name	Abbreviation
Epithelial derived neutrophil activating peptide 78 or CXCL5	ENA-78
Basic fibroblast growth factor	bFGF
Granulocyte colony stimulating factor	G-CSF
Granulocyte macrophage colony stimulating factor	GM-CSF
Interferon gamma	IFN-gamma
Interleukin 1alpha	IL-1alpha
Interleukin 2beta	IL-1beta
Interleukin 1 receptor antagonist	IL-1ra
Interleukin 2	IL-2
Interleukin 4	IL-4
Interleukin 5	IL-5
Interleukin 6	IL-6
Interleukin 8	IL-8
Interleukin 10	IL-10
Interleukin 17	IL-17
Monocyte chemotactic protein 1 or CCL2	MCP-1
Macrophage inflammatory protein 1alpha or CCL3	MIP-1a
Macrophage inflammatory protein 1beta or CCL4	MIP-1b
Regulated upon activation, Normal T-cell expressed or CCL5	RANTES
Tumor necrosis factor alpha	TNF-alpha
Thrombopoietin	TPO
Vascular endothelial growth factor	VEGF
Stromal derived factor 1alpha	SDF-1alpha
